# Associations of Community Water Fluoridation with Caries Prevalence and Oral Health Inequality in Children

**DOI:** 10.3390/ijerph14060631

**Published:** 2017-06-13

**Authors:** Han-Na Kim, Jeong-Hee Kim, Se-Yeon Kim, Jin-Bom Kim

**Affiliations:** 1 Department of Dental Hygiene, College of Health Sciences, Cheongju University, 298, Daesung-ro, Cheongwon-gu, Cheongju 28503, Korea; hnkim@cju.ac.kr; 2 Department of Preventive and Community Dentistry, School of Dentistry, Pusan National University, 49, Busandaehak-ro, Mulgeum-eup, Yangsan, Gyeongsangnam-do 50612, Korea; laura65@hanmail.net (J.-H.K.); secan00@naver.com (S.-Y.K.); 3 BK 21 PLUS Project, School of Dentistry, Pusan National University, Yangsan 50612, Korea

**Keywords:** dental caries reduction, children, dental caries, inequality, oral health, community water fluoridation, social inequalities

## Abstract

This study aimed to confirm the association between the community water fluoridation (CWF) programme and dental caries prevention on permanent teeth, comparing to a control area, neighbouring population without the programme, and verifying whether the programme can reduce the socio-economic inequality related to the oral health of children in Korea. Evaluation surveys were conducted among 6-, 8-, and 11-year-old children living in Okcheon (CWF) and neighbouring Yeongdong (non-CWF, control area) towns in South Korea. Data on monthly family income, caregiver educational level, and Family Affluence Scale scores were evaluated using questionnaires that were distributed to the parents. The effectiveness of CWF in caries reduction was calculated based on the differences in decayed, missing, and filled teeth and decayed, missing, and filled tooth surfaces indices between the two towns. The data were analysed using logistic regression and univariate analysis of variance. Both 8- and 11-year-old children living in the CWF area had lower dental caries prevalence than those living in the non-CWF community. Differences in dental caries prevalence based on educational level were found in the control area but not in the CWF area. Socio-economic factor-related inequality in oral health were observed in the non-CWF community. Additionally, 8- and 11-year-old children living in the CWF area displayed lower dental caries prevalence in the pit-and-fissure and smooth surfaces than those living in the non-CWF community. These results suggest that CWF programmes are effective in the prevention of caries on permanent teeth and can reduce oral health inequalities among children. The implementation of CWF programmes should be sustained to overcome oral health inequalities due to socio-economic factors and improve children’s overall oral health.

## 1. Introduction

Among the top 10 diseases paid for in an outpatient clinic by the Korean National Health Insurance System in 2012, pulpitis and root apical lesion ranked third and ninth as the most frequently claimed items, respectively. The total cost of both diseases was more than US $66.6 million [1,2]. The majority of dental and periapical tissue diseases are caused by severe dental caries progression. Consequently, dental caries is regarded as a major health and economic burden among Korean people.

Community water fluoridation (CWF) is the addition of a controlled amount of fluoride at a constant concentration of 0.8 ppm to the public water supply with the intent of preventing dental caries among the population [3]. The dental caries prevalence in several countries with advanced oral health systems has reduced since the first introduction of water fluoridation in 1945 [4,5,6,7]. In Korea, the first CWF programme was initiated in 1981 at Jinhae City. It was later expanded to include 32 local areas and 36 water treatment plants to cover 8.9% of the Korean population in 2002. Through this programme, the oral health in this population has considerably improved, with the mean decayed, missing, and filled teeth (DMFT) index among Korean children decreasing steadily since the 1980s [8,9]. Currently, the areas implementing the CWF programme have been reduced to 20 local areas and 24 water treatment plants, which only cover 6.7% of the Korean population. This decrease is believed to be due to the lack of understanding among many community residents on how water fluoridation programmes work and the failure of public health officials to adequately educate these residents on the benefits of these programmes [10].

The DMFT index is one of the simplest and most commonly used indices in epidemiological surveys of dental caries. It quantifies the dental health status based on the number of carious, missing, and filled teeth. In contrast, the DMFS index is based on the number of carious, missing, and filled tooth surfaces. Both indices have been used for more than 50 years and are well established as key measures of caries experience in dental epidemiology [11].

Okcheon is located in the Chungcheongbuk-do Province of Korea and is the only suburban area that has been implementing CWF programmes for at least 80% of its total population since 1997. Yeongdong is the neighbouring town of Okcheon that does not implement the CWF programmes. Both towns have similar characteristics in terms of socio-economic status (SES), living conditions, and dental service access. For example, in 2010, the dentist-to-population ratios were 1:3859 and 1:3886 in Okcheon and Yeongdong, respectively. Hence, the caries prevalence between both towns can be suitably compared. Previous studies have reported the caries preventive effects of the CWF programmes [5,7,12,13], although most have reported these effects in the urban areas and not in the suburban areas. Additionally, some studies simply compared caries prevalence between CWF and non-CWF areas without controlling for confounding factors [14] or comparing neighbouring areas. The effectiveness of CWF in caries reduction, therefore, has not yet been assessed by comparing two areas that have similar demographics and other socio-economic variables.

Other studies have reported SES as a risk factor for dental caries [15,16,17] because it can influence the diet and frequency of brushing one’s teeth [18,19]. Furthermore, as the total DMFT index in children decreases, differences in caries reduction based on the SES levels can be observed. However, conceptual and methodological hurdles complicate most attempts to assess the effect of SES on dental caries prevalence among children. Conceptually, whether parental SES should be used as a proxy and, if so, which aspect of SES should be regarded as the most relevant is debatable [20]. Methodologically, parental SES information is difficult to obtain from children. Considering that either complication can result in incorrect data, a new measurement index was developed: the Family Affluence Scale (FAS) [20,21]. The FAS score is determined by choosing a set of items, such as phone, car, and bedroom, that reflected the family expenditure and consumption relevant to family circumstances in the early 1990s in Scotland [22]. To date, little evidence has been presented to confirm the effects of water fluoridation based on socio-economic level using the FAS.

Recently, the oral health of children has improved because of the use of many preventive approaches, such as fluoride toothpaste, sealant treatment, and CWF programmes [23]. Despite the overall decline in dental caries among children, severe dental caries can still be observed in this population. Previous studies reported that racial and community economic factors cause inequality in oral health [24]. CWF can reduce the gap in oral health caused by socio-economic conditions. It is an economic undertaking implemented at a low cost for many people [25], and its implementation is not determined based on the SES of the residents. Some studies showed that water fluoridation could substantially reduce inequalities in dental health [25,26,27] but does not appear to close the gap in oral health among children with different social environments [28].

This study aimed to confirm the association between CWF and dental caries prevention, comparing dental caries prevalence between CWF and neighbouring community without the programme, and confirm whether the programme can reduce oral health inequalities related to socio-economic factors among Korean children. 

## 2. Methods

### 2.1. Participants

The ethical approval for the study was obtained from University of Yangsan-Pusan Hospital Institutional Review Board (IRB No. 05-2012-034). Written informed consent was received from the parents of all participating children prior to data collection. Permission to conduct the study was obtained from the directors of public health centres and principals of the selected schools.

Participants were recruited for a cross-sectional study from the primary schools in the CWF and non-CWF neighbouring areas. The two biggest primary schools from the most populous area in Okcheon were selected as the experimental sample schools using a convenient cluster sampling method. In a similar manner, the three biggest primary schools from the most populous area in Yeongdong were selected as the control sample schools.

The study population consisted of children aged 6, 8, and 11 years old. In this study, 11-year-old children were selected although the World Health Organization (WHO) guidelines recommended the assessment of DMFT index among 12-year-old children as the representative age group. In Korea, children between the ages of 6 and 11 years are enrolled in elementary school. Therefore, the oral examination in this study was performed among elementary school students, and the analysis was conducted for 11-year-old children. The number of all children available for both oral examination and questionnaire survey was 1411, which included 660 non-CWF and 751 CWF participants. Participants who did not answer the questionnaires on either FAS level, monthly mean family income, or parental educational level were excluded from the analysis (number of excluded participants: 98).

### 2.2. Oral Examination

In accordance with the WHO criteria for oral health surveys [29], oral examinations of the participants were conducted under a blue-white portable examination light (Kimscope HeadLight^TM^ SLL-05, Kimscope, Korea) in October 2011. The raw data on the dental caries prevalence and presence of fissure-sealed teeth were collected through oral epidemiological examinations performed by three dentists who had training courses for inter-examiner reliability and participated in the 2010 Korean National Oral Health Survey. Prior to the field survey in 2010, calibration training was administered to 20 students, which was conducted at a dental school. The agreement degree for the caries was 0.90 for kappa. Examinations were directly recorded onto a specially designed datasheet by trained assistants.

### 2.3. Questionnaires

Prior to oral examinations, a parental questionnaire survey was conducted to collect SES information, such as educational level, monthly family income, and FAS level. The classification criteria for monthly family income were presented as selected items based on the general salary of Korean workers. Subsequently, the monthly family income was divided into four categories: <200 10,000 won (KR)/month, 200–300 10,000 won (KR)/month, 300–400 10,000 won (KR)/month, and >400 10,000 won (KR)/month. These values are equivalent to the following upon conversion to US dollars: ≤$1743, $1744–$2615, $2616–$3478, and ≥$3478, respectively. The monthly family income was then categorized as low, mid-low, mid-high, or high level.

The questionnaire consisted of four pages that include oral examination recording sheets, SES questions, and permission form. Many respondents are reluctant to respond to socio-economic questions, particularly on income and educational level. The FAS index was developed to compensate for this tendency. The modified FAS questions developed by Boyce et al. [20] considered four items as a measure of family wealth: number of cars owned, owning an unshared bedroom, number of family trips taken, and number of computers owned. A score of 1 is given for each available item. Unless the number of each available item is greater than 1, the appropriate corresponding number is given. Thus, the typical FAS scores range from 0 to 9 per participant, with a higher FAS value implying a higher SES.

### 2.4. Statistical Analysis

IBM SPSS Version 21.0 was used for data analysis. Both the DMFT and DMFS indices were calculated. Subsequently, dental caries increments were calculated as the differences between CWF and non-CWF areas in terms of DMFT, DMFS, pit-and-fissure DMFS, and smooth surface DMFS indices prior to the adjustment of each variable for confounding factors. The tooth surfaces were categorized into two groups by taking into consideration the participants’ ages: pit-and-fissure and smooth surfaces. Pit-and-fissure surfaces included the occlusal surfaces on premolars and molars, buccal surfaces on lower molars, and lingual surfaces on upper molars [28]. Meanwhile, the smooth surfaces included the remaining other surfaces on the molars and all tooth surfaces on the anterior teeth.

The associations between caries prevalence and CWF programme implementation based on socio-economic factors were determined using χ^2^-test and independent sample *t*-test. Furthermore, logistic regression was presented as odds ratio (OR) and 95% confidence intervals (CI) against the reference category of each factor to determine their effects on dental caries. Regression analysis was used to confirm the β coefficients, which can explain the relationship between the variables and DMFT indices. The DMFT index distribution among the 11-year-old children from the CWF and non-CWF areas were shown using FAS to compare the reduction in public health inequalities in terms of dental caries at each FAS level. A difference was considered to be statistically significant at *p* < 0.05. 

## 3. Results

A total of 322 males and 305 females in the non-CWF area and 353 males and 333 females in the CWF area were included in the analysis. The 6-, 8-, and 11-year-old age groups in both areas were similarly distributed (Table 1). 

The most commonly reported monthly family income in the non-CWF (25.7%) exceeded US $3478, whereas that in the CWF area (30.5%) was between US $1744 and US $2615. Almost 95% of householders from both areas graduated from primary school. The non-CWF area had a slightly higher number of families with high FAS level than the CWF area (*p* = 0.019). However, the overall FAS level distribution in both areas was similar. Additionally, the non-CWF area displayed a higher percentage of persons with dental sealants than the CWF area (*p* < 0.001) (Table 2). 

Table 3 shows the mean scores of caries experience based on the DMFT, DMFS, pit-and-fissure DMFS, and smooth surface DMFS scores, which were calculated using analysis of covariance (ANCOVA) after adjusting for sex, monthly family income, householder educational level, FAS score, and number of fissure-sealed teeth. The 8- and 11-year-old children in the CWF area showed significantly lower mean DMFT scores than those in the non-CWF area. For example, the 8-year-old children living in the CWF area (0.15) had significantly lower DMFT scores than those living in the non-CWF area (0.56) (*p* < 0.001). Moreover, the 11-year-old children in the CWF area also showed lower mean DMFT scores (0.86) than those in the non-CWF area (1.43) (*p* < 0.001). However, the difference in the DMFT scores among 6-year-old children in both areas was not statistically significant. The 8-year-old children in the CWF area (0.13) demonstrated significantly lower pit-and-fissure DMFS scores than those in the non-CWF area (0.52) (*p* < 0.001). A similar instance was also observed among the 11-year-old children in both areas (CWF and non-CWF areas: 0.75 and 1.27, respectively; *p* < 0.001). Additionally, 8-year-old children in the CWF area (0.09) had significantly lower smooth surface DMFS scores than those in the non-CWF area (0.0.28) (*p* = 0.004). Similarly, the 11-year-old children in the former also showed significantly lower smooth surface DMFS scores than those in the latter (CWF and non-CWF areas: 0.57 and 0.93, respectively; *p* = 0.008).

Table 4 displays the OR of caries experience based on the results of the logistic regression analysis. In the non-CWF area, age, sex, householder educational level, and number of fissure-sealed tooth surfaces were associated with caries experience. However, in the CWF area, only age was confirmed to be the variable related to caries experience.

Table 5 presents the DMFT scores based on SES-related factors. Children living in non-CWF area with lower householder educational level tend to have more dental caries than those with householders who were college/university graduates or higher (β = 1.03, *p* < 0.001). The number of fissure-sealed teeth among children living in the non-CWF area was inversely associated with the DMFT score (β = −0.22, *p* < 0.001).

**Figure 1:**
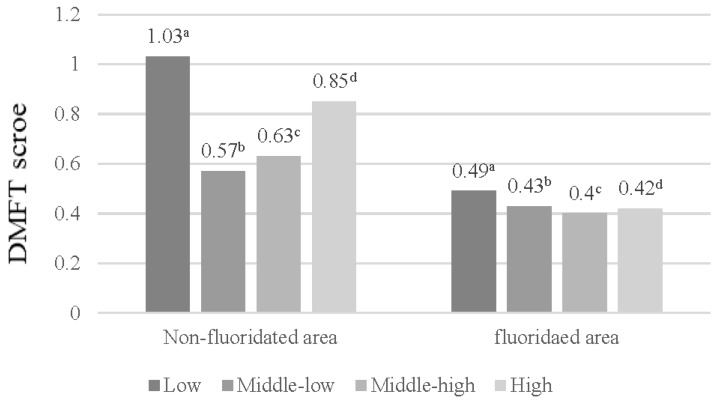
Distribution of decayed, missing, and filled teeth (DMFT) scores based on the monthly family income. The scores were calculated from the univariate analysis of variance: ^a^ Covariates appearing in the model are evaluated at the following values: age = 8.58; ^b^ Covariates appearing in the model are evaluated at the following values: age = 8.27; ^c^ Covariates appearing in the model are evaluated at the following values: age = 8.66; ^d^ Covariates appearing in the model are evaluated at the following values: age = 8.89.

**Figure 2:**
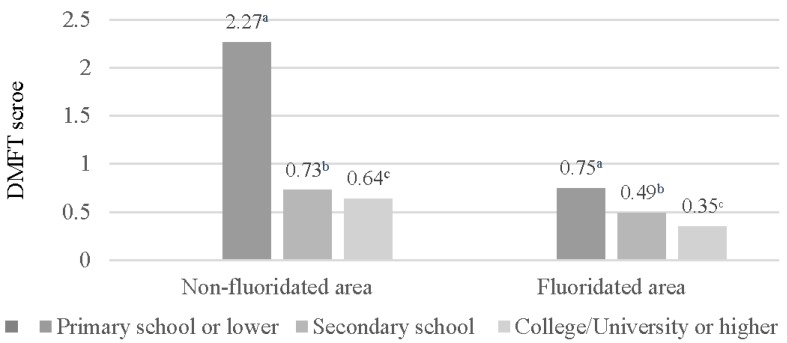
Distribution of decayed, missing, and filled teeth (DMFT) scores based on the householder educational levels (year). The scores were calculated from the univariate analysis of variance: ^a^ Covariates appearing in the model are evaluated at the following values: age = 9.93; ^b^ Covariates appearing in the model are evaluated at the following values: age = 8.61; ^c^ Covariates appearing in the model are evaluated at the following values: age = 8.40.

**Figure 3:**
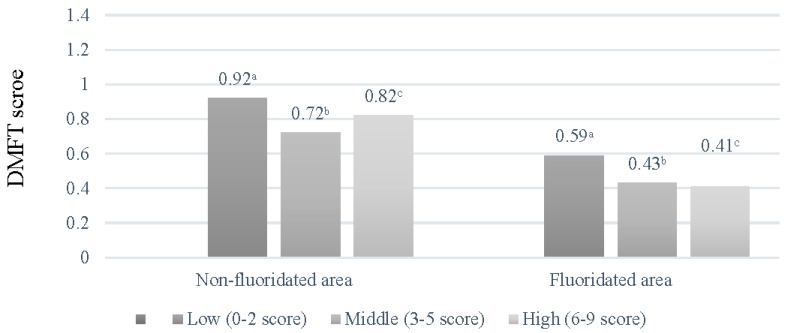
Distribution of decayed, missing, and filled teeth (DMFT) scores based on the Family Affluence Scale scores. The scores were calculated from the univariate analysis of variance: ^a^ Covariates appearing in the model are evaluated at the following values: age = 8.53; ^b^ Covariates appearing in the model are evaluated at the following values: age = 8.56; ^c^ Covariates appearing in the model are evaluated at the following values: age = 8.63.

Figure 1 shows that no difference in the DMFT scores based on monthly family income were observed in the CWF area, in contrast to the non-CWF area, where differences in the DMFT scores were noted. Figure 2 and Figure 3 display the DMFT score distribution based on the householder educational and FAS levels. The DMFT scores could be assumed to be higher in groups with lower householder educational and FAS levels. Additionally, the differences in these variables were greater in the non-CWF area than in the CWF area.

**Figure 4:**
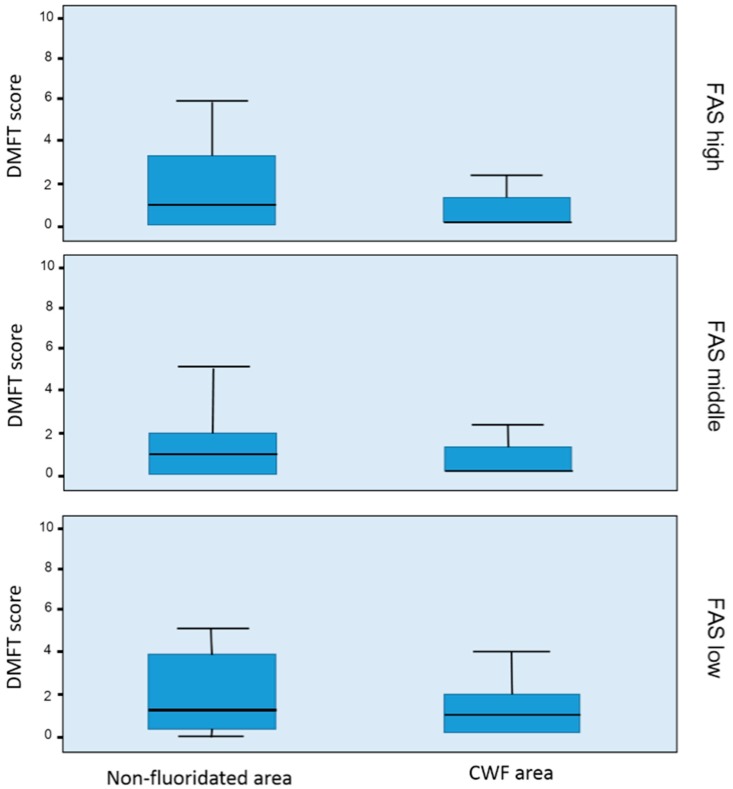
Box plots for the decayed, missing, and filled teeth (DMFT) scores of 11-year-old children based on the Family Affluence Scale (FAS) levels.

The DMFT scores of 11-year-old children were compared using box plots (Figure 4), which indicated that the DMFT score distribution was divided based on the three FAS levels. The range of the box plots in the CWF area was more compact than that in the non-CWF area. The caries experience of the lowest one third in the non-CWF area had wider boxplots than that in the CWF area. The widening gap was evident between both areas. The lowest FAS groups displayed larger reduction in caries experience than the other FAS groups (Figure 1).

## 4. Discussion

Several caries preventive interventions have been introduced in the recent years. Some, which are often used in dental clinics, focus on individual caries activity, such as caries management based on risk assessment or detection and prevention of early dental caries [30,31,32]. These preventive interventions have advantages and disadvantages as well, such that they are notably conducted only for patients who visit dental clinics. The frequency of dental visits can be viewed as a function of SES and may reflect inequalities with regard to oral health. Kim and Jeon [33] demonstrated that the DMFT index of 12-year-old Korean children decreased between 2000 and 2010 and the inequality of oral health had increased, which they showed using a Lorenz curve. If so, then the number of caries-free children is increasing. However, numerous children still have significant caries experience. CWF is one of the representative public oral health programmes, which provide oral health benefits to citizens at a low cost regardless of the SES level. Furthermore, we evaluated the reduction in caries prevalence by comparing the DMFT and DMFS scores between both neighbouring areas.

The DMFT index could not be used to express the oral health status of 6-year-old children because this population has a mixed dentition. No noteworthy results in the caries prevalence and OR of this age group were observed. Additionally, the permanent teeth of 6-year-old children have not been exposed long enough to fluoride to be of any benefit to the comparison in the CWF area.

Caries prevalence among 8- and 12-year-old children is higher in girls than in boys based on the results of the 2012 Korean National Oral Health Survey [9]. Therefore, adjusting for sex proportions during the calculation of caries increments on permanent teeth was necessary to reveal the differences in caries experience between the CWF and non-CWF areas.

Song et al. [31] reported that the DMFT index of Korean adults was higher in groups with lower educational level and lower in groups with higher family income level. Furthermore, the low educational level of a mother was associated with an increase in sugar intake among her children [34]. Park et al. [35] indicated that the socio-economic environments of adolescents also act as health and oral health behavioural factors.

Although children and adolescents are aware of socio-economic inequalities and inequitable opportunities [23], many adolescents cannot accurately report their parents’ jobs or educational levels, much less their incomes [19,36]. FAS was developed with a view towards overcoming these problems. Although many prior studies have attempted to incorporate SES factors using FAS [36,37,38,39], we could not find any correlation between FAS and caries experience of children in this study. 

A previous systematic review on the prevention of dental caries using water fluoridation reported 35% and 26% reduction in decayed, missing, and filled deciduous and permanent teeth, respectively, compared with the median control group mean values [40]. However, the majority of the included studies (71%) were conducted prior to 1975 and the widespread use of fluoride toothpaste [41]. In this study, we documented the caries prevalence in a CWF area, Okcheon town, and compared it with that in a neighbouring non-CWF area, Yeongdong town. The majority of children living in both areas was widely assumed to use fluoridated toothpaste [41], and major confounding variables, such as sex, FAS score, householder educational level, monthly family income, and number of fissure-sealed teeth, were adjusted using ANCOVA. The reduction in caries prevalence on permanent teeth among suburban children living in fluoridated areas has been estimated to be approximately 50%. These findings support the use of water fluoridation to prevent dental caries even in children who use fluoride toothpaste and the expansion of such programmes into other suburban areas in Korea. Additionally, Kang et al. [42] reported the effects of water fluoridation programmes that have been implemented for seven years on caries prevalence. Their results showed that the reduction in caries prevalence among 8- and 11-year-old children before and after the programme were 64.4% and 55.5%, respectively. Similar to the results of Kang et al.’s study [42], the reduction in caries prevalence as a result of water fluoridation programmes in Okcheon may be approximately 50% as well. Surprisingly, we found low pit-and-fissure DMFS scores among 8- and 11-year-old children who lived in the CWF area. Given that caries reduction is expected for smooth surfaces, this result is surprising for pit-and-fissure surfaces, which was adjusted for the number of fissure-sealed teeth.

No differences in the OR of caries experience based on SES indicators and number of fissure-sealed tooth surfaces were found among children living in the CWF area. Additionally, oral health inequality was not observed among participants from the CWF area. These findings add to the evidence demonstrating the associations between fluoridated drinking water and dental caries experience [43]. However, in the non-CWF area, a difference in the rate of caries experience based on age, sex, householder educational levels, and number of fissure-sealed tooth surface was observed. Furthermore, in the non-CWF area, children whose householder had lower educational level were more likely to have caries than those whose householder had higher educational attainment (OR = 2.66), and children who had less fissure-sealed teeth were also more likely to have caries than those who have more fissure-sealed teeth. Such results have been reported elsewhere [32,44]. Recently, van der Tas et al. [45] observed that the maternal educational level was the most important indicator of association between socio-economic position and caries. Parents or fosterers with low educational level would have worse health literacy and dietary and oral health behaviour and lower health service utilization than those with higher educational attainment. Therefore, CWF might reduce the caries prevalence associated with these problems among children.

The DMFT score boxplots of 11-year-old children that were divided based on the three DMFT levels were wider in the non-CWF area than in the CWF area throughout the three FAS levels. Moreover, the lines that signify the mean DMFT in each box were higher in the former than in the latter. These findings suggest not only that the children in the non-CWF area had higher mean DMFT than those in the CWF area but also that more children had severe caries in the former than in the latter. These results indicate that water fluoridation programmes can reduce the mean DMFT and number of children with severe caries throughout all the FAS levels. Moreover, regardless of FAS levels, this study confirms the reduction of caries prevalence through water fluoridation. Peres et al. [44] also reported that water fluoridation is an effective method in reducing the inequalities in dental caries distribution. 

Notwithstanding the strengths of this study, a number of potential limitations merit consideration. Despite using ANCOVA to control for related variables, other confounding factors that might affect the dental caries prevalence should still be considered, especially the duration of the participants’ exposure to fluoridated water. If the information on the residency period of the participants in each area were available, then these data could be used to further refine the assessment on the effectiveness of water fluoridation. Additionally, the examination of the effect of non-fluoridated toothpaste use on caries prevalence was not possible, although the numbers were expected to be relatively small. A cohort or further studies that control for these other confounding factors are needed to confirm the association between water fluoridation and dental caries. The reliability of the associations between FAS and SES in the analysis of oral health survey data also needs to be confirmed. Considering that the DMFT scores of children are highly skewed, models for count variables, such as Poisson and negative binomial analysis, might be more appropriate in the analysis. 

Generally, the DMFT index of 12-year-old children as a representative age group can be used to compare the caries prevalence among children from varying nations. In this study, oral health examinations were not conducted among 12-year-old children; instead, the examinations were performed among 11-year-olds on behalf of this age group. Therefore, representative ages are lacking that allow for comparison of the children’s oral health. The caries prevalence rate was higher than expected. The caries experience rates of 8- and 11-year-old children were 12.3% and 34.6%, respectively, in the CWF area (not shown in the tables), and 27.6% and 54.2%, respectively, in the non-CWF area. Therefore, the conventional regression model could be used to estimate the effect of a caries prevention programme on caries prevalence. However, the purpose of this study was to determine whether the dental caries experience was reduced in both regions, rather than studying the occurrence of caries or determining the number of new caries that did not occur. Several previous studies that reported on the caries prevalence rate following the implementation of prevention programmes compared the differences in the DMFT index.

## 5. Conclusions

This study shows that CWF is associated with the reduction in caries prevalence in the era of widespread fluoride toothpaste use. Caries reduction in the pit-and-fissure and smooth surfaces of 8- and 11-year-old children who lived in the CWF area was observed. No differences in caries experience due to other variables, especially socio-economic factors, were noted in the CWF area. Additionally, oral health inequalities caused by SES have not been identified in the CWF area. The lowest FAS groups displayed larger reduction in caries prevalence among 11-year-old children expressed through box-plot distribution than the other groups. Addressing other confounding factors for dental caries, such as duration of exposure to fluoridated water, sugar consumption, and social disadvantage between CWF and non-CWF areas, is also necessary.

## Figures and Tables

**Table 1:** Number of participants in areas with and without community water fluoridation (CWF).

Age	Non-CWF Area			CWF Area		
	Total	Male	Female	Total	Male	Female
Total	627	322	305	686	353	333
6	164	79	85	221	104	117
8	203	115	88	211	101	110
11	260	128	132	254	148	106

**Table 2:** Characteristics and oral health status of the participants.

Variables	Category	Non-CWF Area		CWF Area		*p*-Value *
		N	%	N	%	
Monthly family income	Low	160	25.5	153	22.3	0.031
	Middle-low	149	23.8	209	30.5	
	Middle-high	157	25.0	174	25.4	
	High	161	25.7	150	21.9	
Householder educational level	Primary school or lower	33	5.3	43	6.3	0.154
	Secondary school	308	49.1	301	43.9	
	College/university graduate or higher	286	45.6	342	49.9	
Family Affluence Scale	Low (0–2)	30	4.8	55	8.0	0.019
	Middle (3–5)	342	54.5	388	56.6	
	High (6–9)	255	40.7	243	35.4	
Percentage of children with dental sealant		627	49.1	686	37.0	<0.001

* Results of the chi-square test.

**Table 3:** Decayed, missing, and filled teeth (DMFT), decayed, missing, and filled tooth surfaces (DMFS), pit-and-fissure DMFS, and smooth surface DMFS scores adjusted for confounding factors.

Caries Experience	Age	Non-CWF Area		CWF Area		*p*-Value ^†^
		Mean *	SE	Mean *	SE	
DMFT ^a^	6	0.13	0.04	0.13	0.03	0.940
	8	0.56	0.06	0.15	0.06	<0.001
	11	1.43	0.10	0.86	0.10	<0.001
DMFS ^b^	6	0.16	0.06	0.19	0.05	0.769
	8	0.79	0.09	0.22	0.08	<0.001
	11	2.20	0.17	1.31	0.17	<0.001
Pit-and-fissure DMFS ^c^	6	0.08	0.04	0.11	0.03	0.536
	8	0.52	0.05	0.13	0.05	<0.001
	11	1.27	0.09	0.75	0.09	<0.001
Smooth surface DMFS ^d^	6	0.09	0.03	0.08	0.03	0.890
	8	0.28	0.04	0.09	0.04	0.004
	11	0.93	0.09	0.57	0.09	0.008

**^a^** Denotes the mean number of decayed, missing, and filled teeth; ^b^ Denotes the mean number of decayed, missing and filled tooth surfaces; ^c^ Denotes the mean number of decayed, missing, and filled teeth on the pit-and-fissure surfaces; ^d^ Denotes the mean number of decayed, missing, and filled tooth surfaces on the smooth surfaces; * Indicates estimated marginal means; ^†^ Calculated using independent sample t-test between the non-CWF area and CWF areas after adjusting for sex, monthly family income, householder educational level, Family Affluence Scale score, and number of sealed teeth.

**Table 4:** Odds ratio (OR) of caries experience adjusted for related variables.

Variables	Category	Non-CWF Area	CWF Area
		OR (95% CI) *	OR (95% CI) *
Age		1.66 (1.50, 1.84)	1.51 (1.35, 1.69)
Sex (ref = male)		1.72 (1.17, 2.52)	1.50 (0.99, 2.28)
Monthly family income (ref = high)	Low	0.61 (0.35, 1.06)	1.38 (0.72, 2.63)
	Middle-low	1.04 (0.59, 1.83)	1.63 (0.86, 3.12)
	Middle-high	1.41 (0.78, 2.54)	2.03 (1.00, 4.13)
Householder educational level (ref = primary school or lower)	Secondary school	0.96 (0.63, 1.45)	1.13 (0.71, 1.82)
	College/university graduate or higher	2.66 (1.08, 6.54)	1.65 (0.76, 3.61)
Family Affluence Scale (ref = high)	Middle	0.73 (0.48, 1.10)	1.00 (0.60, 1.62)
	Low	1.02 (0.38, 2.75)	1.72 (0.78, 3.77)
Number of fissure-sealed tooth surface		0.80 (0.70, 0.91)	1.09 (0.97, 1.22)

Nagelkerke R-square = 0.30 and 0.19 for non-CWF area and CWF areas, respectively; * Results of the logistic regression analysis adjusted for age, sex, living area, monthly family income, householder educational level, and Family Affluence Scale level; bold letters indicate significant differences.

**Table 5:** Univariate analysis of variance for decayed, missing, and filled teeth scores.

Parameter	Category	Non-CWF Area			CWF Area		
		B	SE	*p*	B	SE	*p*
Intercept		−1.1	0.26	<0.001	−0.86	0.18	<0.001
Age		0.29	0.03	<0.001	0.15	0.02	<0.001
Sex (ref = male)	Male	−0.39	0.10	<0.001	−0.22	0.07	0.004
Monthly family income (ref = high)	Low	0.04	0.16	0.82	0.03	0.13	0.83
	Middle-low	−0.17	0.15	0.26	0.08	0.11	0.48
	Middle-high	−0.24	0.14	0.10	0.00	0.11	1.00
Householder educational level (ref = college/university graduate or higher)	Primary school or lower	1.03	0.25	<0.001	0.12	0.17	0.46
	Secondary school	0.04	0.11	0.71	0.09	0.08	0.26
Family Affluence Scale	Low	−0.08	0.26	0.76	0.19	0.16	0.22
	Middle	−0.08	0.11	0.48	0.01	0.08	0.94
	High	0	–	–	0	–	–
Number of fissure-sealed teeth		−0.22	0.03	<0.001	−0.01	0.02	0.71

R-square for non-CWF area = 0.26 (adjusted R-square = 0.25); R-square for CWF area = 0.11 (adjusted R-square = 0.10); Bold letters indicate significant differences.

## References

[1] Health Insurance Review and Assessment Service (2012). 2012 Health Insurance Statistical Report.

[2] Fejerskov O., Kidd E. (2009). Dental Caries: The Disease and its Clinical Management.

[3] Pizzo G., Piscopo M.R., Pizzo I., Giuliana G. (2007). Community water fluoridation and caries prevention: A critical review. Clin. Oral Investig..

[4] Bunelle J., Carlos J. (1990). Recent trends in dental caries in US children and the effect of water fluoridation. J. Dent. Res..

[5] Loh T. (1996). Thirty-eight years of water fluoridation—The Singapore scenario. Community Dent. Health.

[6] Rock W., Gordon P., Bradnock G. (1981). Caries experience in West Midland school children following fluoridation of Birmingham water in 1964. Caries of first permanent molars. Br. Dent. J..

[7] Spencer A., Slade G., Davies M. (1996). Water fluoridation in Australia. Community Dent. Health.

[8] Han D.H., Kim J.B., Park D.Y. (2010). The decline in dental caries among children of different ages in Korea, 2000–2006. Int. Dent. J..

[9] Ministry of Health and Welfare (2013). The Report of Korean National Oral Health Survey in 2012.

[10] Kim D., Bae K., Kim J. (2005). The residents’ knowledge and attitude and factors related to the approval of adjusted water fluoridation program in Gimhae. J. Korean Acad. Dent. Health.

[11] Anaise J.Z. (1984). Measurement of dental caries experience-modification of the DMFT index. Community Dent. Oral Epidemiol..

[12] Newbrun E. (1989). Effectiveness of water fluoridation. J. Public Health Dent..

[13] Lewis D., Banting D. (1994). Water fluoridation: Current effectiveness and dental fluorosis. Community Dent. Oral Epidemiol..

[14] Shin H.J., Yang D.K., Han D.H., Lee S.M., Bae K.H., Kim J.B. (2008). Public health Dentistry: The effect of 5-year community water fluoridation program on dental caries prevention of permanent teeth in the western area of Jeju, Korea. J. Korean Acad. Oral Health.

[15] Cho H.J., Lee H.S., Paik D.I., Bae K.H. (2014). Association of dental caries with socioeconomic status in relation to different water fluoridation levels. Community Dent. Oral Epidemiol..

[16] Reisine S.T., Psoter W. (2001). Socioeconomic status and selected behavioural determinants as risk factors for dental caries. J. Dent. Educ..

[17] Taani D.Q. (2002). Relationship of socioeconomic background to oral hygiene, gingival status, and dental caries in children. Quintessence Int..

[18] Chopra A., Rao N.C., Gupta N., Vashisth S., Lakhanpal M. (2015). The predisposing factors between dental caries and deviations from normal weight. N. Am. J. Med. Sci..

[19] Levin K., Currie C. (2009). Inequalities in toothbrushing among adolescents in Scotland 1998–2006. Health Educ. Res..

[20] Currie C., Molcho M., Boyce W., Holstein B., Torsheim T., Richter M. (2008). Researching health inequalities in adolescents: The development of the Health Behaviour in School-Aged Children (HBSC) family affluence scale. Soc. Sci. Med..

[21] Boyce W., Torsheim T., Currie C., Zambon A. (2006). The family affluence scale as a measure of national wealth: Validation of an adolescent self-report measure. Soc. Indic. Res..

[22] Currie C.E., Elton R.A., Todd J., Platt S. (1997). Indicators of socioeconomic status for adolescents: The WHO Health Behaviour in School-aged Children Survey. Health Educ. Res..

[23] Petersen P.E., Lennon M.A. (2004). Effective use of fluorides for the prevention of dental caries in the 21st century: The WHO approach. Commun. Dent Oral Epidemiol..

[24] Do L.G., Spencer A.J., Slade G.D., Ha D.H., Roberts-Thomson K.F., Liu P. (2010). Trend of income-related inequality of child oral health in Australia. J. Dent. Res..

[25] Griffin S.O., Jones K., Tomar S.L. (2001). An economic evaluation of community water fluoridation. J. Public Health Dent..

[26] Riley J.C., Lennon M.A., Ellwood R.P. (1999). The effect of water fluoridation and social inequalities on dental caries in 5-year-old children. Int. J. Epidemiol..

[27] Lee M., Dennison P.J. (2004). Water fluoridation and dental caries in 5- and 12-year-old children from Canterbury and Wellington. N. Z. Dent. J..

[28] Lalloo R., Jamieson L.M., Ha D., Ellershaw A., Luzzi L. (2015). Does fluoride in the water close the dental caries gap between Indigenous and non-Indigenous children?. Aust. Dent. J..

[29] World Health Organization (1997). Oral Health Surveys: Basic Methods.

[30] Brunelle H.A., Carlos J.P. (1982). Changes in the prevalence of dental caries in U.S. school children. J. Dent. Res..

[31] Song K.B., Choi Y.H., Hong S.J., Kim J.B. (2003). Dental caries prevalence in relation to socioeconomic factors and dental health behaviors among Korean adults. J. Korean Acad. Oral Health.

[32] Maheswari S.U., Raja J., Kumar A., Seelan R.G. (2015). Caries management by risk assessment: A review on current strategies for caries prevention and management. J. Pharm. Bioallied Sci..

[33] Kim C.S., Jeon J.E. (2013). Trends in oral health inequality in 12-year-old Korean children: A study using the Gini coefficient. J. Korean Acad. Oral Health.

[34] Ahmed N.A., Åstrøm A.N., Skaug N., Petersen P.E. (2007). Dental caries prevalence and risk factors among 12-year old schoolchildren from Baghdad, Iraq: A post-war survey. Int. Dent. J..

[35] Park Y.D., Patton L.L., Kim H.Y. (2010). Clustering of oral and general health risk behaviors in Korean adolescents: A national representative sample. J. Adolesc. Health.

[36] Duncan G.J., Brooks-Gunn J., Klebanov P.K. (1994). Economic deprivation and early childhood development. Child. Dev..

[37] Kim J.W., So W.Y., Kim Y.S. (2012). Association between asthma and physical activity in Korean adolescents: The 3rd Korea Youth Risk Behavior Web-based Survey (KYRBWS-III). Eur. J. Public Health.

[38] Park E. (2010). A comparative study of youth health risk behaviors by region: Focused on metropolitan areas, medium sized and small city areas, and rural areas. J. Korean Acad. Nurs..

[39] Jung S.H., Tsakos G., Sheiham A., Ryu J.I., Watt R.G. (2010). Socio-economic status and oral health-related behaviours in Korean adolescents. Soc. Sci. Med..

[40] Iheozor-Ejiofor Z., Worthington H.V., Walsh T., O’Malley L., Clarkson J.E., Macey R., Alam R., Tugwell P., Welch V., Glenny A.M. (2015). Water fluoridation for the prevention of dental caries. Cochrane Library.

[41] Kim J.Y., Lee J.H., Park H.K., Kim E.K., Kim J.B. (2003). User rate of fluoride-containing toothpaste in Ulsan Metropolitan City. J. Korean Acad. Oral Health.

[42] Kang E.J., Shin S.C., Lyoo Y.J., Park K.S., Lee S., Min H.H. (2005). 7 years study on the caries prevention effect of water fluoridation at Ok-cheon county. J. Korean Acad. Oral Health.

[43] Slade G.D., Davies M.J., Spencer A.J., Stewart J.F. (1995). Associations between exposure to fluoridated drinking water and dental caries experience among children in two Australian states. J. Public Health Dent..

[44] Peres M.A., Antunes J.L., Peres K.G. (2006). Is water fluoridation effective in reducing inequalities in dental caries distribution in developing countries? Recent findings from Brazil. Soz. Praventivmed..

[45] Van der Tas J.T., Kragt L., Elfrink M.E.C., Bertens L.C.M., Jaddoe V.W.N., Moll H.A., Ongkosuwito E.M., Wolvius E.B. (2017). Social inequalities and dental caries in six-year-old children from the Netherlands. J. Dent..

